# Functional Index Questionnaire: structural validity study in Brazilian patients with anterior knee pain

**DOI:** 10.1590/1516-3180.2024.0153.R1.07032025

**Published:** 2025-06-27

**Authors:** André Pontes-Silva, Almir Vieira Dibai-Filho, Flávio de Oliveira Pires, Carlos Eduardo Girasol, Gabriel Gardhel Costa Araujo, Plínio da Cunha Leal, José Djalma Arrais, Cid André Fidelis-de-Paula-Gomes, Christian Emmanuel Torres Cabido

**Affiliations:** I Departamento de Fisioterapia, Programa de Pós-Graduação em Fisioterapia, Universidade Federal de São Carlos (UFSCar), São Carlos (SP), Brazil.; II Departamento de Educação Física, Programa de Pós-Graduação em Educação Física, Universidade Federal do Maranhão (UFMA), São Luís (MA), Brazil.; III Departamento de Educação Física, Programa de Pós-Graduação em Educação Física, Universidade Federal do Maranhão (UFMA), São Luís (MA), Brazil.; IV Postgraduate Program in Reabilitação e Desempenho Funcional, Universidade de São Paulo (USP), Ribeirão Preto (SP), Brazil.; V Departamento de Educação Física, Programa de Pós-Graduação em Educação Física, Universidade Federal do Maranhão (UFMA), São Luís (MA), Brazil.; VI Departamento de Medicina, Programa de Pós-Graduação em Educação Física, Universidade Federal do Maranhão (UFMA), São Luís (MA), Brazil.; VII Departamento de Fisioterapia, Programa de Pós-Gradução em Ciências da Reabilitação, Universidade Nove de Julho (Uninove), São Paulo (SP), Brazil.; VIII Departamento de Fisioterapia, Programa de Pós-Gradução em Ciências da Reabilitação, Universidade Nove de Julho (Uninove), São Paulo (SP), Brazil.; IX Departamento de Educação Física, Programa de Pós-Graduação em Educação Física, Universidade Federal do Maranhão (UFMA), São Luís (MA), Brazil.

**Keywords:** Patellofemoral pain syndrome, Knee, Osteoarthritis, Pain, Reproducibility of results, Musculoskeletal disorders, Consensus-based Standards for the Selection of Health, Measurement Instruments (COSMIN), Chronic Pain, Patient-Reported Outcome Measures (PROMs)

## Abstract

**OBJECTIVE:**

To assess the Functional Index Questionnaire (FIQ) structure using confirmatory factor analysis (CFA) in Brazilian patients with anterior knee pain.

**METHODS:**

Brazilian patients of both sexes (n = 100), aged ≥ 18 years, with anterior knee pain for at least 3 months were included. Eligible participants completed an online form that collected personal and clinical data as well as responses to the assessment tools. We used CFA and the following fit indices: chi-square/degrees of freedom (DF), comparative fit index (CFI), Tucker–Lewis index (TLI), root mean square error of approximation (RMSEA), and standardized root mean square residuals (SRMR).

**RESULTS:**

The majority of the respondents were women, young adults, overweight, with incomplete higher education, were physically active, and had pain in sitting or squatting positions. The mean duration of pain was 38.24 months, and the mean pain intensity was 4.54 points. The model fit indices were as follows: χ^2^/DF = 2.08, TLI = 0.978, CFI = 0.969, RMSEA = 0.104, and SRMR = 0.077. Therefore, the one-dimensional structure with eight items yielded an RMSEA value above the 0.08 cutoff point, suggesting a poorer fit and more residual error than is acceptable for a well-fitting model. Using the modification indices within the CFA, we observed a correlation between Items 2 (climbing up two flights of stairs [16 steps]) and 6 (climbing up four flights of stairs [32 steps]) and Items 3 (squatting) and 4 (kneeling), indicating the similarity in the response pattern for these items. After adding these correlations to the model, we obtained improved fit indices (χ^2^/DF = 1.51, TLI = 0.990, CFI = 0.985, RMSEA = 0.072, and SRMR = 0.061).

**CONCLUSION:**

This version of the FIQ should be used with caution, as the unidimensional model demonstrates substantial residuals, mainly because of item redundancy.

## INTRODUCTION

The use of patient-reported outcome measures to assess anterior knee pain is recommended.^1,2^ Consequently, several clinical assessment instruments have been reported in the literature.^3-6^ The Functional Index Questionnaire (FIQ) is a widely used outcome measure for assessing functional limitations in patients with anterior knee pain.^3-6^ This instrument was first developed by Stratford and Heuff6 and subsequently validated by Chesworth et al. ^5^ It is easy to administer, and its reliability and validity have been documented in patients with anterior knee pain. Moreover, patients with anterior knee pain haven chosen the FIQ as the easiest questionnaire to complete among other outcome measures. ^1^

Currently, the FIQ has been cross-culturally adapted and translated into Persian^4^ and Brazilian Portuguese. ^3^ Negahban et al.^4^ examined test-retest reliability, internal consistency, construct validity, and factor analysis to determine the number of underlying factors and the items that load on each factor. Cunha et al.^3^ tested the internal consistency, ceiling and floor effects, construct validity, reproducibility, and responsiveness. However, the Brazilian version of the FIQ^3^ did not assess the internal structure of the FIQ as recommended in the guidelines.^7^

Therefore, this study aimed to assess the structure of the FIQ using confirmatory factor analysis (CFA) in Brazilian patients with anterior knee pain. We hypothesized that the unidimensional 8-item model of the FIQ would demonstrate adequate fit. 

## METHODS

### Study design and recruitment

 This study has a cross-sectional design and investigated the structural validity of the FIQ.^3^ Data collection for the study was conducted using an online form (Google Forms, Mountain View, California) in rehabilitation clinics and gyms in São Luís, northeast of Brazil. 

 This study was approved by the Research Ethics Committee of the Universidade Federal do Maranhão (report number 3.995.226), and the guidelines were in accordance with the Declaration of Helsinki (i.e., all experiments were performed in accordance with the relevant guidelines and regulations). All respondents freely participated in the study and signed an informed consent form. 

### Sample size and eligibility

 The minimum sample size considered in this study was 100 participants, as recommended by the Consensus-Based Standards for the Selection of Health Measurement Instruments (COSMIN). ^7^ We included participants of both sexes, sedentary or active, between the ages of 18 and 60 years, who reported anterior knee pain for at least 3 months. ^8^ We adopted the following exclusion criteria: history of trauma, fracture, or acute injury to the knee joint; knee surgery; use of analgesics in the past seven days; physiotherapy treatment for anterior knee pain in the past 3 months; and presence of other chronic pain.^9^

### Evaluation tools

 We used the numerical pain rating scale (NPRS) to characterize participants’ pain intensity.^10^ It is a unidimensional scale from 0 to 10 points, where 0 is 'no pain' and 10 is the 'worst pain imaginable' with adequate validity for Brazilians.^10^ In addition to the NPRS and an initial assessment (covering personal and anthropometric characteristics of the sample), we applied the FIQ, a scale adapted for Brazilian Portuguese^3^ with eight items and three response options for each item ('unable to do' = 0 points, 'can do with a problem' = 1 point, and 'no difficulty' = 2 points). The total score ranges from 0 ('complete inability to perform everyday life activities') to 16 ('no problems performing everyday life activities'). 

### Statistical analysis

 We performed a descriptive analysis and presented the data as means and standard deviations, or as relative and absolute frequencies. We used CFA to identify the best structure of the FIQ using R Studio (Boston) and the lavaan and semPlot packages. A polychoric matrix and robust diagonally weighted leastsquares (RDWLS) extraction method were applied.^11,12^

 We considered adequate values of fit indices for the following cutoff values: χ^2^/degree of freedom (DF) < 3; comparative fit index (CFI) and Tucker–Lewis index (TLI) > 0.90; and root mean square error of approximation (RMSEA) and standardized root mean square residuals (SRMR) < 0.08.^13,14^ Factor loadings were considered adequate when >0.40,^15^ and we used the modification indices to identify redundancy or discrepancies in the model. 

## RESULTS

 A total of 100 participants participated in this study. Among them, 65% were young women (31.76 [12.28]), overweight (body mass index > 25 kg/m^2^), incomplete higher education (38%), and physical activity practitioners (64.4%) (**Table 1**). We observed that most participants presented with pain in the sitting (82%) or squatting (67%) positions, with a mean pain duration of 38.24 months and a mean pain intensity of 4.54 points. A similar distribution of unilateral pain was observed on the right (33%), left (36%), and bilateral (31%) sides (**Table 2**). 

**Table 1:** Personal and anthropometric characteristics of the sample (n = 100)

Characteristics	Mean (standard deviation) or %
Age (years)	31.76 (12.28)
Body mass (kg)	71.15 (15.24)
Stature (m)	1.65 (0.08)
Body mass index (kg/m^2^)	25.79 (4.39)
Sex	
*Male*	65%
*Female*	35%
Education	
*Incomplete primary education*	1%
*Primary education*	1%
*Incomplete secondary education*	1%
*Secondary education*	12%
*Incomplete graduate*	38%
*Graduate*	26%
*Incomplete postgraduate*	5%
*Postgraduate*	16%
Lower limb dominance	
*Right*	78%
*Left*	13%
*Both*	9%
Physical activity	
*Yes*	35%
*No*	65%

**Table 2:** Clinical variables

Pain characteristics	Mean (standard deviation) or %
Sitting (yes)	82%
Squatting (yes)	67%
Running (yes)	61%
Jumping (yes)	61%
Up or downstairs (yes)	65%
Time of pain (months)	38.24 (49.97)
Knee in pain (dominance)	
*Right*	*33%*
*Left*	*36%*
*Both*	*31%*
FIQ (score, 0–16)	11.24 (3.4)
NPRS (score, 0–10)	4.54 (1.99)

FIQ: Functional Index Questionnaire; NPRS: numerical pain rating scale.

 Regarding the internal structure of the Brazilian version of the FIQ, the following fit indices were observed in the CFA: χ^2^/DF = 2.08, TLI = 0.978, CFI = 0.969, RMSEA = 0.104 (90% confidence interval = 0.059–0.149), and SRMR = 0.077. Therefore, the one-dimensional structure with eight items presented RMSEA values above the 0.08 cutoff point, indicating a greater number of residuals than is acceptable for a model. 

**Figure 1:**
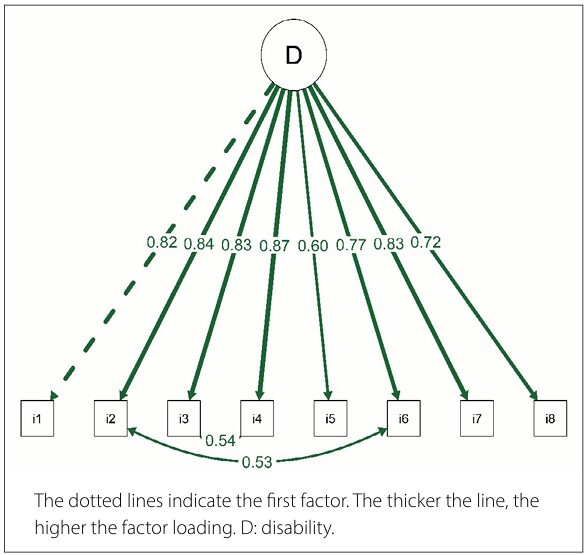
Path diagram of the 8-item Functional Index Questionnaire (FIQ).

 Using the fit indices within the CFA, we observed a high correlation between the following items: items 2 (climbing up two flights of stairs [16 steps]), 6 (climbing up four flights of stairs [32 steps]), 3 (squatting), and 4 (kneeling). This indicates a similarity in the response patterns for these items. We added these correlations to the model (Figure 1) and found the following suitable fit indices: χ^2^/DF = 1.51, TLI = 0.990, CFI = 0.985, RMSEA = 0.072 (90% confidence interval = 0.000–0.124), and SRMR = 0.061. 

## DISCUSSION

 We aimed to assess the structure of the FIQ using CFA in Brazilians with anterior knee pain and hypothesized that the unidimensional 8-item model of the FIQ is adequate. Our results, rejecting the hypothesis, showed that the onedimensional structure with eight items presented more residuals than acceptable for the model. In addition, the high correlation between the items indicated a similarity in the response patterns for these items. Because of redundancy, this version of the FIQ should be used with caution and complemented by other similar assessment tools, such as the Kujala Score or the Anterior Knee Pain Scale. 

Reports on FIQ are limited. It was first reported at the Annual Congress of the Canadian Physiotherapy Association (Canada)^6^ and has only been culturally adapted into two versions (Brazilian3 and Persian^4^ ). Using the Persian version of the FIQ, Negahban et al.^4^ performed factor analysis on a sample of 100 patients with patellofemoral pain syndrome. As a limitation of this study,^4^ the authors recommended the assessment of responsiveness in subsequent studies. However, no researchers have investigated this measurement property in a Persian sample. Subsequently, in the Brazilian version of the FIQ, Cunha et al.^3^ investigated internal and external responsiveness, as recommended by Negahban et al.^4^ However, unlike the Persian version,^4^ the Brazilian version of the FIQ^3^ did not examine the internal structure through factor analysis. 

 This is the first study to assess the structure of the FIQ using CFA in Brazilian patients with anterior knee pain. However, some limitations of this study must be recognized to accurately interpret the results. First, the study population had a mean body mass index of 25.79 kg/m^2^, and whether these results would vary in lean or obese Brazilians needs to be ascertained. Second, the study population had chronic pain with a mean time of 38.24 months and a mean pain level of 4.54 (NPRS) at the time of evaluation; hence, future studies should try to replicate our results in patients with acute pain needs. Third, the limited number of studies on this topic makes it difficult to compare our results. 

 Future studies should consider the redundancy of the items identified here and modify the instrument by proposing other functional activities that may cover different aspects of the lower limb function commonly performed by individuals with anterior knee pain, thus increasing the evaluative capacity of the FIQ. 

## CONCLUSION

 This version of the FIQ should be used with caution because the unidimensional model of the questionnaire presents large residuals, mainly because of the redundancy among the items. 
